# Production Methods, Biological Activity and Potential Application Prospects of Astaxanthin

**DOI:** 10.3390/foods14122103

**Published:** 2025-06-15

**Authors:** Fajian Ren, Chaolong Rao, Qiwen Xiang, Jiayu Wen, Qiuju Dai, He Li, Jiayu Liang, Yan Chen, Cheng Peng

**Affiliations:** 1 School of Public Health, Chengdu University of Traditional Chinese Medicine, Chengdu 611137, China; renfajian0072@163.com (F.R.); raocl@cdutcm.edu.cn (C.R.); xiangqw@stu.cdutcm.edu.cn (Q.X.); wenjiay0126@163.com (J.W.); 15892249375@163.com (Q.D.); 18880472102@163.com (H.L.); 18784511980@163.com (J.L.); 2 State Key Laboratory of Traditional Chinese Medicine Resources in Southwest China, Chengdu 611137, China

**Keywords:** astaxanthin, ketocarotenoid, source, biological activity, mechanisms, application prospects

## Abstract

Astaxanthin (AST), a ketocarotenoid, is prevalent in aquatic life forms. AST has a variety of health-promoting effects, such as anti-oxidation, anti-cancer, eye protection, anti-inflammatory, immune regulation, skin care, anti-diabetes, neuroprotection, etc. It holds significant potential for applications in healthcare products, food additives, pharmaceuticals, cosmetics, and aquaculture. The production capacity of AST limits its wide application to a certain extent. The instability and safety risks associated with the chemical synthesis of AST have led to increased interest in its biosynthetic pathway. In this paper, the synthesis pathway, biological activity, and application prospects of AST were reviewed. To enhance the market accessibility of AST, investigating innovative synthesis techniques and its emergent biological effects is crucial.

## 1. Introduction

Natural bioactive compounds are becoming more and more popular. The ideal traits are high bioactivity, bioavailability, health benefits, and safety, with low production and processing costs increasing their attractiveness. Astaxanthin (AST) (3,3-dihydroxy-β, β-carotene-4,4-dione) is a natural bioactive substance found in microalgae, crustaceans, fish, and birds. Current research indicates that AST possesses amphiphilic properties [1,2]. AST is a carotenoid characterized by the chemical formula C_40_H_52_O_4_, a molecular mass of 596.85 Da, and a density of 1.081 g/L. AST concentrations differ significantly across aquatic species and tissues, with European trout meat containing 6 mg/kg, Japanese trout meat 25 mg/kg, and cultured Atlantic salmon meat 6–8 mg/kg [3]. *Haematococcus pluvialis* (*H. pluvialis*) contains the highest natural concentration of AST, able to accumulate up to about 5%, Only AST isolated from *H. pluvialis* is considered as safe for human ingestion [4,5]. In recent years, the demand for natural AST in the global market has continued to increase [6], prompting researchers to continuously optimize the extraction and biosynthesis of AST.

**Figure 1:**
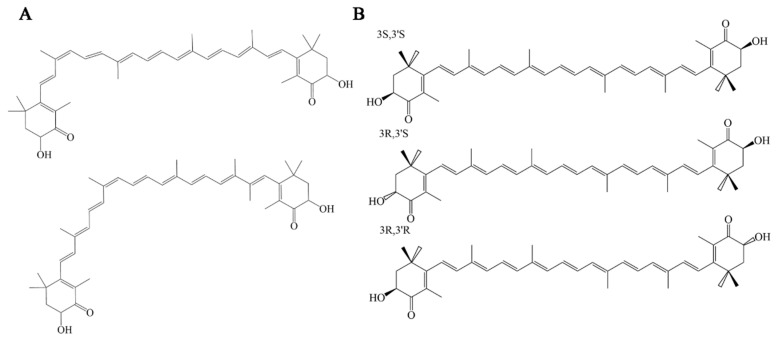
(**A**) Structures of 9-cis-AST and 13-cis-AST; (**B**) structures of (3S,3′S), (3R,3′R), and (3R,3′S) all-trans-AST.

The molecular structure of AST is similar to β-carotene and other carotenoids, composed of two β-ionone ring systems. Different from other carotenoids, AST is composed of two β-ionone ring systems. The ionone ring has hydroxyls at 3,3′ and ketones at 4,4′ positions. They enhance AST’s polarity, enabling it to neutralize free radicals and significantly improving its cell membrane permeability [7,8]. AST’s dual ionone rings, with elongated polyene cores, efficiently capture high-energy electrons, contrasting with β-carotene’s 11 carbon–carbon diunsaturated bonds. AST possesses two isomers, trans and cis, attributed to its carbon–carbon double polyunsaturated bonds (Figure 1) [9]. The cis-AST encompasses both 9-cis and 13-cis configurations (Figure 1A). Two stereocenters located at the C-3 and C-3′ sites in all-trans AST yield three stereoisomers: (3S, 3′S), (3R, 3′R), and (3R, 3′S) (Figure 1B). All-trans AST exhibits greater stability compared to cis AST, suggesting it is the predominant natural form of AST [10]. Moreover, 3S, 3′S-AST is the most effective isomer for human use [11,12].

At present, the AST in the market is mostly of synthetic production; this chemically synthesized AST, due to its inherent safety issues, is usually not directly used in the fields of human medicine and health supplements. A small fraction of industrial AST comes from sources like the alga *H. pluvialis* or other AST-producing organisms [4]. AST outperforms other carotenoids, such as zeaxanthin, lutein, and carotene, in terms of biological activity thanks to its distinct molecular properties. In recent years, numerous studies have explored its effects across both animal and human trials, solidifying its reputation as a potent bioactive compound. AST possesses multiple biological activities, including antioxidant [13,14], anticancer [15,16,17], anti-diabetes [18,19], anti-inflammatory [20,21], immune regulation [22], and protection of the nervous, cardiovascular, and dermal systems [23,24,25]; these characteristics have attracted widespread attention. Studies suggest that the rising recognition of natural AST’s wellness advantages and security will fuel a 16.2% composition annual rate of growth, hitting USD 339.88 million by 2027. The biological activity and mechanisms of AST are shown in Table 1.

This review first summarizes the common methods of extracting AST at present, thereby offering insight to investigative peers. Then, the detailed mechanisms of AST in anti-oxidation, anti-cancer, eye care, anti-inflammation, regulation of immunity, skin care, anti-diabetes, and neuroprotection are reviewed. This research offers a solid foundation for expanding AST’s commercial applications across the food, pharmaceutical, cosmetic, and nutraceutical industries. The findings also establish crucial scientific groundwork for future exploration and practical use of AST.

## 2. AST Production Methods

Numerous methods have been investigated for producing AST. These include extraction from crustacean byproducts (e.g., krill, shrimp, crab), the farming of natural organisms such as microalgae, bacteria, and yeast, and the chemical manufacture of substances. Direct extraction from crustacean waste results in low yield and high costs, while cultivating natural producers is also associated with relatively high production expenses [65,66]. In contrast, chemical synthesis offers better cost-effectiveness. Moreover, 95% of AST on the market is produced through chemical synthesis [67]. According to reports, the chemical synthesis of AST can be categorized into two main methods: full synthesis and semi-synthesis. In full synthesis, industrial chemicals are used as starting materials to produce AST through a series of chemical reactions, with the Witting reaction being a hallmark of this process. On the other hand, semi-synthesis involves utilizing natural carotenoids like lutein as the base material. The most renowned semi-synthesis technique begins with lutein, which undergoes isomerization in the presence of a base to form zeaxanthin. This conversion is followed by a reaction with 1,2-propanediol as the solvent and potassium hydroxide as the catalyst at a temperature of 110 degrees Celsius for 168 h. Under the influence of iodine and sodium bromate, zeaxanthin is then directly oxidized into AST [68]. Although chemical synthesis can yield large quantities of AST quickly, due to the fact that chemical synthesis easily produces a mixture of AST stereoisomers (left-handed: racemic: right-handed 1:2:1), and some of these components cannot be found in nature, the activity exhibited is difficult to determine, thus presenting certain safety risks. Moreover, compared to natural AST, chemically synthesized AST also has shortcomings such as poor stability and a complex production process [69]. Hence, the utilization of AST in chemical synthesis is increasingly discouraged, particularly in sectors like pharmaceuticals, cosmetics, and food production [69,70]. This paper mainly introduces the research progress of direct extraction and biosynthesis of AST in recent years. The advantages and disadvantages of different production methods of AST are shown in Table 2.

### 2.1. Biosynthesis of AST

Microalgae, yeast, bacteria, and plant cultivation enables the synthesis of AST [67,79]. As far as we know at present, animals cannot synthesize AST on their own. The animal foods containing AST (such as salmon and crustaceans) accumulate AST by consuming microorganisms. It is reported that during the process of microbial production of AST, isopentenyl pyrophosphate (IPP) and dimethylallyl pyrophosphate (DMAPP) are the early precursors for microbial synthesis of AST. These compounds further lead to the synthesis of geranylgeranyl diphosphate (GGPP), which is the origin of carotenoids. Two GGPP molecules can combine to produce phytoene, and phytoene is the universal precursor for all carotenoids, including AST [65].

*H. pluvialis* is the leading natural AST producer. The culture consists of two stages: green and red, with AST accumulation occurring during the red stage [16]. However, due to the slow growth rate of *H. pluvialis* and the relatively long production stage, it is vulnerable to pollution in the production stage [65], which compels investigators to concentrate on the choice and enhancement of lineages and refine the present manufacturing methodology [80,81]. Chen et al. [82] found that a *H. pluvialis* mutant generated through high-light low-temperature plasma (LTP) mutagenesis efficiently produces AST, with transcriptome and metabolic analyses conducted to investigate the underlying reasons for this efficiency. The mutant enhances CO_2_ utilization efficiency and facilitates AST precursor formation by reducing fructose-1,6-bisphosphatase (FBP) expression while increasing the expression of enzymes such as malate dehydrogenase (MDH), phosphoenolpyruvate carboxylase (PEPC), and ribulose bisphosphate carboxylase/oxidase (Rubisco) activator enzyme (RCA). In addition, in *H. pluvialis*, the biosynthesis of AST and fatty acids requires a large amount of ATP and nicotinamide adenine dinucleotide phosphate (NADPH). The genetically altered strain reduces light-induced oxidative stress by adjusting the chlororespiratory process and boosting concentrations of accessory pigments like lutein, β-carotene, and AST. This adaptation not only maintains robust photosynthetic efficiency but also promotes the production of AST. Yu et al. [83] optimized the culture conditions of *H. pluvialis*, and added 15% walnut shell extract (WSE) to the *H. pluvialis* medium. Finally, the contents of AST and lipids were increased by 77.57% and 23.39%, respectively. The primary mechanism involves WSE modulating the Light–oxygen–voltage–sensing Histidine Kinase, which in turn influences gene transcription related to AST and fatty acid biosynthesis pathways, as well as the ROS-mediated antioxidant system, ultimately regulating AST and lipid biosynthesis. Besides *H. pluvialis*, *Coelastrum* sp. HA-1 is a microalga capable of accumulating lipids and AST. Most natural ASTs are esterified by fatty acids in microalgae to prevent oxidation. Thanks to the fact that only slightly more than half of the AST molecules in HA-1 got the oleaginous treatment, AST accumulation was not as high as in *H. pluvialis.* In an attempt to increase HA-1 production, Liu et al. [77] observed that spraying HA-1 with a small amount of linoleic acid (LA) and a squirt of ethanol can supercharge the esterification process. When exposed to 35 μM LA, the concentrations of AST esters and total AST (TA) surged to 3.82-fold and 2.18-fold of the control values, respectively. Introducing 3% (*v*/*v*) ethanol further boosted the metabolic shift from oleic acid (OA) to LA, driving AST ester and TA levels even higher—reaching 2.42 and 1.61 times the basal levels.

*Xanthophyllomyces dendrorhous* (*X. dendrorhous*) is a promising microorganism for enhancing AST production more efficiently than the *H. pluvialis*-based method [84]. Hara et al. [70] optimized the culture conditions for direct production of AST by *X. dendrorhous* yeast. They used the peel extract of ponkan (*Citrus poonensis*) as a nutrient for the growth of *X. dendrorhous*. Finally, they found that the peel extract of ponkan can provide abundant carbon sources, nitrogen sources, minerals, and other activators for *X. dendrorhous* and AST; the arabinose contained therein serves as a crucial metabolic precursor substance which significantly promotes the proliferation of the bacteria and the biosynthesis of AST, thereby effectively enhancing the yield of AST. The optimum addition amount of ponkan peel extract was 40 g/L.

*Escherichia coli* (*E. coli*) itself cannot accumulate AST, but it can be produced by genetic engineering [66]. The researchers [85] selected the *E. coli* strain CAR026 as the go-to candidate for the synthesis of AST. They obtained a genetically stable Escherichia coli strain by coordinating the expression of β-carotene ketolase (CrtW) and β-carotene hydroxylase (CrtZ) and increasing the copy number of the lycopene cyclase (CrtY) gene; CrtY encodes lycopene cyclase, which catalyzes the β-cyclization of lycopene to generate β-carotene, a critical intermediate for downstream reactions. In fermentation trials, the microorganism yielded 0.88 g of AST per liter and 5.9 milligrams per gram of dry cell mass (DCW). Nonetheless, during the process, proteins CrtZ and CrtW tend to misfold, thereby impeding the yield. To counter this issue, the gene responsible for the molecular chaperone, groES-groEL, is modulated to maximize AST output. The strain Gro-46, after a 60 h batch fermentation period, delivered an impressive AST output of 1.18 g per liter.

Among the existing AST production methods, the cost-effectiveness of AST synthesized by chemical synthesis is the highest [86]. Its use in human consumption is restricted due to possible residues of intermediates and by-products from the synthesis process [65,69]. For the newly proposed green process supercritical fluid extraction (SFE), non-heat treatment such as ultrasonic-assisted extraction (UAE), pulsed electric field (PEF), high-pressure treatment (HPP), and other technologies, the reaction temperature is lower, the operating conditions are mild, and the damage to the active ingredients is less, which is suitable for the extraction of heat-sensitive substances such as AST. Studies show that CO_2_ is abundant in sources and low in cost, and it can be used as an extraction medium in a supercritical state to achieve efficient separation. Supercritical carbon dioxide (SFE-CO_2_) extraction efficiently isolates lipophilic compounds, including lipids and antioxidant carotenoids like α-carotene, β-carotene, β-cryptoxanthin, free AST, and esterified AST, from shrimp waste. However, the selectivity of the extracted compounds is not easy to determine, and the recovery rate of AST is relatively low. Enhancing solute solubility and yield requires the use of suitable modifiers, co-solvents, or high pressure [87,88]. In the UAE context, it prompts the lipid oxidation and hydrolysis process. Consequently, PEF pretreatment is necessary prior to utilizing UAE for oil extraction. At the same time, HPP also has similar shortcomings. The extraction process fails to fully inactivate oxidases like peroxidase and polyphenol oxidase, leading to oxidation [89]. Tintchev et al. [90] discovered that high-pressure treatment of smoked salmon leads to AST oxidation, with its degradation linked to metmyoglobin reduction through unidentified mechanisms. For the biosynthesis of AST, the growth rate of microalgae is slow, the biomass is low, the culture is usually less than 15 g/L for about one month, and it is easily polluted, which limits the production speed and titer of AST. Certain non-indigenous microorganisms, such as specific yeasts and *E. coli*, often reach biomass concentrations surpassing 100 g/L within the initial days of fermentation. These microorganisms are ideal for genetic engineering and industrial AST production, significantly enhancing AST yield [65]. In genetic or metabolic engineering, it is crucial to develop straightforward, precise, and efficient methods for screening mutants or engineered bacteria that enhance AST production, alongside establishing more advanced genetic engineering tools [91]. In short, the biosynthesis method is green and sustainable. Microorganism-produced AST is anticipated to increasingly supplant chemically synthesized and naturally extracted AST in the near future.

### 2.2. AST Extraction and Purification

The traditional extraction methods of AST (such as the extraction methods using solvents like ethyl acetate, acetone, ethanol, etc., and the oil extraction method using vegetable oil as the medium) rely on the solubility of the solvent for AST [71,92]. The extraction efficiency is closely related to the polarity, solubility, and intermolecular forces of the solvent. However, these methods face pre-treatment challenges such as dehydration of algal biomass, cell wall disruption (requiring acid/base treatment, low-temperature grinding, mechanical crushing, enzyme lysis, etc., which are energy-intensive and involve multiple steps) [93], the removal of solvents, color fading control, and retention of biological activity. Due to the need for high temperatures, high solvent consumption, or strong mechanical effects under such demanding conditions, AST is prone to oxidation degradation, impurity residue, and structural isomerization, resulting in poor product quality (low purity, high impurities), yield (with a yield of only 30–50% of the theoretical value), and stability (content decreases by 10–20% during storage) [94]. Moreover, when AST is directly dispersed in the oil solvent in the oil extraction method, its half-life is short and stability is poor. It requires the combination of technologies such as spray drying and extrusion to further increase the complexity of the process [72]. Conventional extraction methods often fall short in terms of efficiency, prompting the development of innovative eco-friendly alternatives such as SFE, UAE, PEF, and HPP. These cutting-edge techniques not only streamline the extraction process but also improve yield, elevate product purity, and amplify the bioactive properties of the final extract. Researchers have further optimized these methods for improved application in AST production.

SFE-CO_2_ has gained recognition as an eco-conscious extraction technique for bioactive compounds. Thanks to its low viscosity, exceptional diffusivity, and high density, it efficiently penetrates biomass structures and enhances the dissolution of desired compounds [95]. The solvent’s polarity can influence the extraction of AST. Choosing the appropriate solvent can improve the extraction rate of SFE-CO_2_ [71]. Messina et al. [96] used ethyl fatty acid (TFA) as a solvent to recover AST from shrimp waste by SFE. Finally, it was found that AST had a high yield in TFA extraction, and AST could be significantly enriched to 114.80 ± 1.23 μg/mL by short-path distillation. Furthermore, integrating additional methods with SFE enhances extraction performance. Microwave (MW) pretreatment is a green pretreatment method which can destroy the solid structure of biomass and facilitate the further extraction of target molecules. Nunes et al. [97] combined MW pretreatment with supercritical fluid extraction, which notably decreases extraction time and solvent use, enhancing process efficiency. After experimental exploration, they determined that the best conditions for MW pretreatment were 140 °C, 300 W, 90 s; the optimum conditions of SFE were 500 bar, 40 °C, 13 wt% ethanol, and 30 min. With this method, 1023 μg/g AST was obtained from the crab shell waste of *Cancer pagurus*.

UAE is an effective and environmentally friendly extraction technique. Its performance depends on several key variables, including the solvent-to-solid ratio, processing duration, operating frequency, temperature conditions, ultrasound dispersion patterns, and power intensity [98]. Sharayei et al. [74] enhanced the efficiency of UAE for extracting AST from shrimp shells. They found the best extraction conditions by Box–Behnken design: ultrasonic amplitude 23.6%, time 13.9 min, and temperature 26.3 °C. The extract contained 51.5% AST under these conditions. However, although UAE has many significant advantages, it also has some drawbacks. The UAE method accelerates lipid oxidation, evidenced by rising peroxide value (PV) and thiobarbituric acid reactive substances (TBARS) levels. In order to improve its drawbacks, Gulzar and Benjakul [99] found that preheating and adding tannic acid can reverse these oxidation effects. Pacific white shrimp heads and chests were preheated at 95 °C in a 0.1% tannic acid solution, followed by a 25 min lipid extraction using UAE at 80% ultrasonic amplitude. The final sample exhibited the highest lipid yield, ranging from 13.3 g to 14.1 g/100 g of sample. In addition, PEF pretreatment was helpful to improve the extraction rate of lipids and carotenoids in the head and chest of shrimp because PEF pretreatment could significantly improve the extraction rate of oil and increase the content of bioactive substances in oil through electroporation. PEF is considered to have an inactivation effect on the enzyme, which can reduce lipid oxidation to a certain extent [75,100,101]. Therefore, the production efficiency of AST can be further improved by combining PEF and UAE. Gulzar and Benjakul [100] discovered that using the UAE method following PEF pretreatment enhances the extraction of polyunsaturated fatty acids and carotenoids, such as AST, AST monoester, AST diester, canthaxanthin, and β-carotene.

Compared with traditional methods, HPP can ensure the quality of the extract and is environmentally friendly. Irna et al. evaluated chemical extraction versus HPP for AST recovery from various shrimp species. Extraction was performed at 210 MPa for 10 min using a 7:3 (*v*/*v*) acetone and methanol mixture. The research findings indicated a notable increase in carotenoid levels, jumping from 46.95 μg/mL with conventional chemical extraction to 68.26 μg/mL when HPP was employed. Similarly, AST yields saw a dramatic boost, rising from 29.44 μg/gdw to 59.97 μg/gdw through HPP. These results clearly show that HPP outperforms traditional methods, delivering higher extraction efficiency, better product quality, and faster processing times, making it a superior alternative for industrial applications [76].

In addition to the above methods, the combination of other technologies can also improve the extraction efficiency. As demonstrated by Hamdi et al. [102], AST was extracted from blue crab (*Portunus segnis*) using a n-hexane/isopropanol (50/50) solvent, yielding the highest total carotenoid content with an AST concentration of 5045 μg/g. AST can also be recovered by microbial degradation. Cheong and colleagues [103] employed ‘*Aeromonas hydrophila*’ to break down shrimp shell waste (SSW) for AST extraction. By optimizing key parameters—including a neutral pH of 7.0, 3% sodium glutamate, 1% glucose, and a temperature of 30 °C—they boosted AST recovery by 38%, ultimately reaching a yield of 2.16 U/mL. The production methods of AST are presented in Table 3.

## 3. Bioactivity of AST

### 3.1. Antioxidation

AST demonstrates potent antioxidant capabilities, a hallmark feature of carotenoid compounds, which stems mainly from its unique molecular architecture featuring conjugated double bonds and oxygenated functional groups attached to both its cyclic structures and central AX moiety. The antioxidant mechanisms of AST involves four key aspects: ① reducing the production of reactive oxygen species (ROS)/reactive nitrogen species (RNS) to resist oxidative stress; ② directly scavenging free radicals; ③ boosting intrinsic antioxidant enzymes such as superoxide dismutase (SOD), catalase (CAT), glutathione peroxidase (GSH-Px), and phase II enzymes; ④ promoting antioxidant molecule and gene expression via signaling pathways such as phosphatidylinositol 3-kinase (PI3K)/protein kinase B (Akt), extracellular signal-regulated kinase (ERK), Nuclear factor (erythroid-derived 2)-like 2 (Nrf2)/Kelch-like ECH-associated protein 1 (Keap1), and other signaling pathways.

Studies show that AST’s antioxidant potency is 10 to 100 times stronger than lutein, zeaxanthin, and β-carotene [104,105]. AST’s effectiveness stems from its highly hydrophobic conjugated polyene framework and polar end groups. The central conjugated double bonds donate electrons to neutralize free radicals, transforming them into more stable compounds. This mechanism not only interrupts radical chain reactions but also captures reactive species within its terminal cyclic structure [106]. AST end-rings neutralize ROS, RNS, and free radicals such as superoxide anion, hydrogen peroxide, and singlet oxygen. Polyene strands are exclusively confined to the cellular membrane, while additional constituents are situated both within and beyond it [107]. AST derived from shrimp shells demonstrates antioxidant characteristics, demonstrating scavenging abilities on 1,1-diphenyl-2-picrylhydrazyl (DPPH) and 2,2′-azido-bis(3-ethylbenzothiazoline-6-sulfonic acid) (ABTS) free radicals that are 72 and 220 times greater than those of ascorbic acid, respectively.

Oxidative stress disrupts redox balance, leading to ROS generation [108]. In biological systems, ROS include free radicals (e.g., hydroxyl) and non-radicals (e.g., hydrogen peroxide). Fu et al. [109] established a rodent model of spinal cord ischemia-reperfusion injury by surgically occluding the abdominal aorta, leading to elevated reactive oxygen species (ROS) levels. AST (25 mg/kg) was injected intraperitoneally for 14 days before surgery. It was found that AST could significantly reduce the level of ROS, indicating that AST had a good inhibitory effect on the production of ROS.

Oxidative stress arises when the body’s antioxidant balance is disrupted. SOD, CAT, and GSH-Px are essential antioxidants, pivotal in inhibiting free radicals and acting as the primary line of defense within the antioxidant network [110]. Malondialdehyde (MDA), a primary lipid peroxidation product, acts as a reliable indicator of oxidative stress and quantifies tissue damage from peroxidation [111]. 8-hydroxy-2-deoxyguanosine (8-OHdG) is a significant biomarker for oxidative stress, known for its role in causing DNA damage [112]. Liu et al. [113] developed a framework for assessing oxidative stress in aged Sprague Dawley male rats through the intragastric delivery of D-galactose. After administering AST orally at concentrations of 5, 10, and 15 mg/kg, tests showed a notable boost in antioxidant function. Specifically, AST increased the activity of CAT, SOD, and GSH-Px enzymes by 26%, 30%, and 53%, respectively. At the same time, it effectively reduced MDA concentrations. Serum metabolic profiling and pathway analysis further indicated that AST mitigates oxidative stress via the FOXO pathway, thereby offering neuroprotection. Kim et al. [114] found that administering AST at doses of 5 mg, 20 mg, and 40 mg for three weeks to persistent smokers reduced oxidative damage by inhibiting lipid peroxidation. This was supported by lower MDA and ISP serum levels and higher SOD and CAT enzyme activities, thereby affirming AST’s robust antioxidant capabilities. Abou-Zeid et al. [115] demonstrated that AST (40 mg/kg) mitigates thiacloprid-induced liver injury by decreasing MDA, 8-OHdG, nitric oxide (NO), and inducible nitric oxide synthase (iNOS) levels while enhancing GSH-Px, SOD, and CAT activities.

**Figure 2:**
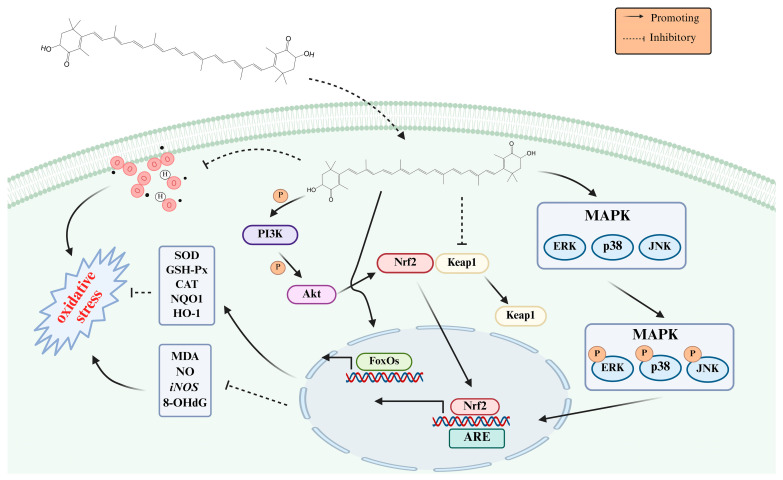
The antioxidant mechanisms of AST.

AST can also regulate various signaling pathways to produce antioxidant effects. The Nrf2/Keap1 pathway is crucial in AST’s resistance to oxidative stress, whether from external or internal sources [116,117]. Keap1 is a cytoplasmic protein that typically binds to and inhibits Nrf2, maintaining its activity in a suppressed state. Upon exposure to ROS, Nrf2 dissociates from Keap1 and engages with antioxidant response elements (ARE), thereby modulating the expression of ARE-dependent antioxidant genes, including enzymes like SOD, CAT, and GSH-Px. In addition to traditional antioxidant enzymes, Nrf2 plays a pivotal role in maintaining cellular redox homeostasis by stimulating multiple protective mechanisms. It promotes the production of glutathione (GSH), activates key detoxification enzymes like glutathione S-transferases (GSTs) and peroxidases, and increases the expression of critical redox regulators including NAD (P) H quinone dehydrogenase 1 (NQO1) and heme oxygenase-1 (HO-1). This multifaceted approach strengthens the cell’s defense system against oxidative stress [118,119,120]. Chen and colleagues [53] conducted a study in which they treated D-galactose-induced aging rats with varying doses of AST (5, 10, and 15 mg/kg). Their findings revealed a notable boost in the activity of key antioxidant enzymes, specifically SOD, CAT, and GSH-Px. Additionally, the researchers observed an up-regulation of Nrf2 expression alongside a corresponding down-regulation of Keap1, suggesting a potential mechanism for the compound’s protective effects, suggested that AST plays an anti-aging role through the Nrf2/Keap1-mediated antioxidant signaling pathway. Nrf2 activation and cell survival regulation depend on the PI3K/Akt pathway, where PI3K phosphorylation initiates Nrf2 signaling and Akt activation [121]. Research showed that AST boosts Nrf2 levels through the PI3K/Akt pathway, triggering phase II enzymes such as NQO1 and HO-1, thereby decreasing ROS formation [122,123]. AST alleviated oxidative stress through regulation of the MAPK signaling route and Nrf2/antioxidant response elements activation [124,125,126]. The antioxidant mechanism of AST is shown in Figure 2.

### 3.2. Anti-Cancer

AST is a potential anti-cancer agent. AST exhibits therapeutic effects on various cancers, including colorectal, melanoma, gastric [127], bladder, liver, blood, lung, oral, and breast cancer [128]. AST targets several molecular pathways in tumor therapy, including P13K/Akt, nuclear factor kappa-B (NF-κB), Nrf2, peroxisome proliferator-activated receptor γ (PPARγ), Bcl2-Associated X Protein (Bax)/Bcl-2, pontin, ERK, c-Jun N-terminal kinase (JNK), Signal transducer and activator of transcription 3 (STAT3), and Mitogen-activated protein kinase (MAPK).

AST demonstrated anti-cancer properties by enhancing antioxidant capacity, reducing oxidative stress [32], and exhibiting anti-inflammatory effects. AST modulates antioxidant enzymes, inflammation, and apoptosis by suppressing the PI3K/Akt pathway, targeting it for cancer therapy [120]. A colon cancer model was established in azoxymethane (AOM)-induced obese C57BL/KsJ-db/db mice. The 200 ppm AST dietary treatment inhibited cell proliferation, reduced oxidative stress, alleviated inflammatory responses, and suppressed NF-κB activation in colon mucosa. Therefore, AST suppresses colon precancerous lesion formation in an obesity-linked colorectal cancer model [31]. Nrf2 alleviates oxidative stress and normalizes cellular redox balance. AST’s anti-tumor and anti-cancer effects occur via Nrf2 pathway activation [129]. AST activates NADPH-dependent oxidase, causing an increase in reactive oxygen species within AGS gastric cancer cells. Among them, 20 μM treatment has the most significant effect and does not cause normal gastric epithelial cell death. Elevated ROS levels trigger phosphorylation of receptor-interacting protein kinase (RIP) 1 and RIP3, leading to mixed-line kinase domain-like protein (MLKL) activation, lactate dehydrogenase (LDH) release, and cell death [32]. Cui et al. [33] found that AST suppressed EC109 oesophageal squamous cell proliferation dose-dependently, with the minimal viability at 50 μM. The mechanisms of AST in oesophageal cancer involves the following: ① enhancing total antioxidant capacity (T-AOC) and SOD while reducing MDA levels to mitigate oxidative stress; ② up-regulating PPARγ, Bax/Bcl-2, and cysteinyl aspartate-specific proteinase 3 (Caspase3) protein levels in oesophageal tissue; and ③ activating downstream apoptotic proteins to initiate the apoptotic pathway.

Modulating pontin expression could be crucial for AST’s anticancer effects. Eliminating pontin, a crucial factor in breast cancer cell growth, can impair breast cancer cells and potentially diminish cancer stem cell (CSC) traits. AST induces G0/G1 arrest and apoptosis in SKBR3 breast cancer cells. AST induces pro-apoptotic effects by down-regulating mutant p53 levels, cleaving poly ADP-ribose polymerase 1 (PARP-1) fragments, increasing Bax expression in a concentration-dependent fashion, and down-regulating Bcl2 levels. It also enhances ERK1/2, JNK, and p38 phosphorylation, increases SOD activity to mitigate ROS, and modulates pontin expression [34]. Ahn et al. [128] showed AST to inhibit the up-regulation of CSC marker genes, such as pontin, Oct4, mutp53, and Nanog, in BT20 and T47D breast cancer cells.

Down-regulation of STAT3 expression is an important part of the anti-cancer effect of AST. STAT3 pathway activation is associated with the development and progression of prostate cancer. In DU145 prostate cancer cells, AST suppresses migration, proliferation, and STAT3 expression (protein/mRNA), while promoting apoptosis [35]. Akt regulates autophagy signaling via the mammalian target of rapamycin (mTOR) pathway modulation. AST suppresses tumor growth and triggers cell death by lowering Akt activity, which in turn inhibits NF-κB, STAT3, and Wnt pathways via decreased p-Akt/Akt levels [36].

AST exhibits potent anticancer effects, including apoptosis induction and suppression of metastasis, invasion, and proliferation. AST demonstrates potential as a new metastasis suppressor in colorectal cancer, significantly inhibiting tumor spread in lab and animal models. Kim et al.’s [37] research employed metastatic colon cancer cell lines CT26 and HCT116, revealing that AST inhibits epithelial-mesenchymal transition (EMT) primarily by up-regulating microRNA (miR)-29a-3p to suppress matrix metalloproteinase (MMP) 2 activity and enhancing miR-200a to inhibit Zinc Finger E-Box Binding Homeobox 1 (ZEB1) expression. The anti-metastatic activity of AST is expressed by negatively regulating the Myelocytomatosis oncogene (MYC) carcinogenic transcription factor. A375 and A2058 melanoma cell models were established. AST (5–125 μg/mL) dose-dependently reduced MMP1, 2, and 9 expressions, inhibiting melanoma cell proliferation and migration. AST induced melanoma cell death by increasing caspase-3/7 levels and halting the cell cycle in the G1 phase [38]. In addition, Shin et al. [39] reported that AST treatment had a stimulating effect on the astrocytic glioma cell line U251-MG and showed an agonistic response. AST treatment at low concentrations (4–8 μM) induced proliferation, whereas high concentrations (20–40 μM) triggered apoptosis. Reduced AST levels may elevate cyclin-dependent kinase (Cdk) 2 and p-Cdk2/3 but suppress p53 expression.

**Figure 3:**
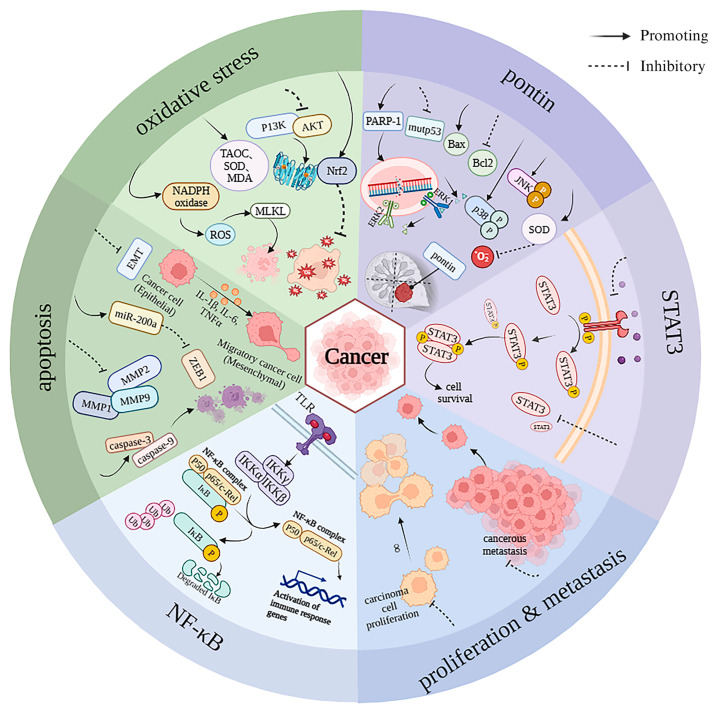
The anti-cancer mechanisms of AST.

AST exhibits anticancer properties and can enhance the efficacy of anti-tumor drugs while mitigating their side effects. In their research, Atalay and colleagues [130] employed MCF-7 breast cancer cells and found that AST bolstered carbendazim’s anticancer prowess by reinforcing the G2/M phase of the cell cycle and lessening the increase in ROS levels triggered by carbendazim. Two NSCLC cell lines, A549 and H1703, were employed to create in vitro models. AST treatment not only inhibited cell viability but also inhibited cell proliferation. AST (2.5–20 µM) reduced Rad51 expression and p-Akt (Ser473) levels in a time- and dose-dependent manner. Furthermore, AST paired with mitomycin C enhances suppression of NSCLC cell proliferation [131]. The anti-cancer mechanisms of AST are shown in Figure 3.

### 3.3. Eye Protection

The eye protection effect of AST is manifested in cataract, amblyopia, retinal diseases, uveitis and ocular surface diseases [132,133]. AST (6 mg/day, for 4 weeks) can alleviate eye fatigue but also reduce darkness, tears, and red eyes [134]. AST’s protective effect on the eye involves molecular targets such as PI3K/Akt, high-mobility group box 1 (HMGB1), p-Akt, hypoxia-inducible factor 1α (HIF1α), X-box binding protein 1 (XBP1), Nrf2, etc.

AST effectively guards against retinal harm or conditions, with its primary protective mechanism stemming from its antioxidant properties. Otsuka and colleagues [40] established both live animal and laboratory cell-based models to study retinal damage caused by ischemia and subsequent reperfusion. Their research showed that when AST is given at a dosage of 100 mg/kg over a four-day period, it markedly reduced the severity of retinal ischemic injury while also causing a noticeable drop in electroretinogram (ERG) response levels. In vitro experiments demonstrated the inhibition of both ROS production and cell death. AST’s protective effects against retinal injury could correlate with the Nrf2 signaling route. AST (50 mg/kg/day) can promote the activation of Nrf2 in retinal ganglion cells (RGCs) of acute glaucoma in mouse model and the activation of its downstream transcriptional regulator HO-1, mediating cell defense against oxidative stress and ROS, thus exerting AST to protect retinal integrity in acute glaucoma mice [135]. Light-emitting diodes (LEDs) are commonly utilized as energy-efficient lighting in daily life; however, their high-intensity short-wave blue light (400–495 nm) can harm the retina. Blue LED exposure induces apoptosis in 661 W photoreceptor cells, while AST (0–100 μM) provides dose-dependent protection. The mechanism involves enhancing Bcl-2/Bax mRNA and protein expression, reducing cellular ROS production, and suppressing oxidative stress biomarkers like nitrotyrosine, 8-OHdG, and acrolein. It also prevents mitochondrial damage, activates the PI3K/Akt pathway, induces Nrf2 nuclear translocation, and up-regulates phase II antioxidant enzymes HO-1 and NQO1 [45]. A mouse model of hyperoxia-induced retinopathy (HIR) was established. Intravitreal (IV) and intraperitoneal (IP) administration of AST can suppress neovascularization and mitigate mitochondrial damage while preventing apoptosis [44]. Moreover, cisplatin (CIS) functions as an antitumor agent, enhancing MDA, iNOS, and 8-OHdG levels while suppressing GSH, thereby leading to retinal cell toxicity. After AST treatment, the levels of related substances can be significantly restored, and the retinal injury caused by CIS can be prevented [136].

AST can improve diabetes-related eye diseases, such as diabetic cataract (DC) and diabetic retinopathy (DR). Oxidative stress in DC is mitigated by antioxidant enzymes and peptides like CAT, SOD, and GSH, which offer protective benefits to lens tissue [41]. When AST was administered at daily doses of either 16 mg/kg or 80 mg/kg over a 24-week period, researchers observed a marked reduction in advanced glycation end products (AGEs), lipid hydrogen peroxide, and MDA levels within lens tissue. Simultaneously, the treatment boosted concentrations of key antioxidant enzymes, including CAT, SOD, and GSH, effectively strengthening the body’s natural defense mechanisms against oxidative stress. HE staining results indicated that AST stabilizes lens epithelial cell morphology, mitigates lens protein degeneration, and inhibits DC progression [41]. AST safeguards ocular protection by blocking vascular endothelial growth factor (VEGF) and neovascularization (ND) effects. DR, a diabetes-related microvascular disorder, involves blood–retinal barrier (BRB) breakdown, where VEGF may worsen disease progression. AST’s anti-VEGF effects may be linked to the suppression of HIF1α and XBP1. The protective impact of AST on DR was examined in vitro using ARPE-19 cells. AST treatment at concentrations of 1, 5, and 10 μM inhibited HIF1α and XBP1 activation and transport triggered by hyperglycemia and cobalt chloride. The treatment also inhibited the movement of retinal pigment epithelial cells in diabetic retinopathy, maintained the integrity of Zona occludin-1 tight junction proteins within the RPE layer, and reduced hyperglycemia- or hypoxia-induced permeability in these cells. In streptozotocin-induced diabetic rats, AST suppression diminishes HIF1α, XBP1, and VEGF expression, subsequently rectifying diabetic retinal layer irregularities [137].

**Figure 4:**
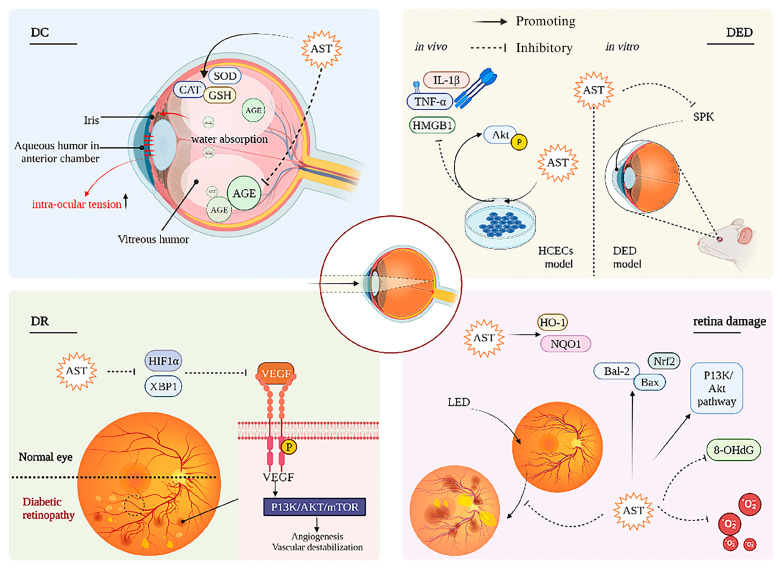
The mechanisms of AST eye protection.

AST may protect against dry eye disease (DED) through mechanisms involving PI3K/Akt. Shimokawa et al. encapsulated AST in liposomes to create high-affinity liposome AST, demonstrating its potential for treating dry eye. An in vitro dry eye cell model demonstrated that affinity liposomes with AST can reduce ROS production in corneal epithelium and enhance lacrimal gland function in aging [138]. A DED rat model was established, and liposome AST can effectively reduce the negative effects of DED-related punctate keratopathy [42]. Li et al. [43] explored how AST enhances DED treatment, using a mouse model in vivo with 5 μM AST and an in vitro human corneal epithelial cells (HCECs) model with 1–10 μM AST. CCK-8 assay showed that AST at concentrations of 1 μM, 2.5 μM, and 5 μM had no cytotoxicity to HCECs. The findings indicated that AST effectively decreased HMGB1 expression in a dose-dependent fashion and suppressed the elevation of inflammatory cytokines TNF-α and IL-1β. The PI3K/Akt pathway may modulate HMGB1 release and AST’s therapeutic impact on DED. The eye-protecting mechanisms of AST are shown in Figure 4.

### 3.4. Anti-Inflammatory

Inflammation is a necessary defensive response initiated by the body to alleviate cellular damage or infection. It can restore tissue homeostasis by regulating cellular and molecular processes. However, persistent inflammatory responses can lead to chronic damage. Recent studies have shown that AST can regulate inflammatory responses through multiple target mechanisms. Compared to other carotenoids, AST has higher anti-inflammatory biological activity [139,140,141].

Oxidative stress and inflammation are predominant components in numerous inflammatory disorders and their associated complications. ROS is naturally produced during normal metabolism and participates in normal wound healing reactions. In oxidative stress, ROS is not properly regulated, and excessive accumulation will increase inflammatory factors leading to chronic inflammation. AST has a strong antioxidant effect and can reduce ROS at the cellular level. It can be used as an antioxidant to participate in the oxidative regulation of wound healing [142]. The mechanism by which AST inhibits inflammation is to activate the Nrf2/Keap1 pathway by reducing ROS content, so that VEGF production inhibits microvascular damage, inhibits inflammation-related factors, and regulates or delays inflammation. It inhibits lipopolysaccharide (LPS)-induced cell death and damage associated with ROS, leading to chronic inflammation [143,144].

AST inhibits MAPK and NF-κB pathways, thereby reducing Tumor necrosis factor-α (TNF-α) and Interleukin (IL-6) cytokine production and release [47]. AST is a primary factor in heart and vascular conditions. AST helps protect the heart from damage by blocking the overactivation of the complement system. This action lowers levels of inflammatory markers like C-reactive protein while preventing harmful buildup of membrane attack complexes. Additionally, it delivers a dual benefit by reducing inflammation and combating oxidative stress [145]. AST suppresses macrophage-induced overexpression of pro-inflammatory cytokine genes [48] and blocks the manifestation of genes activated by cellular inflammation, nuclear factors, and protease secretion [49]. It may impede the MAPK/NF-κB signaling cascade, reduce TNF-α secretion, and safeguard against acute lung inflammatory conditions [47].

**Figure 5:**
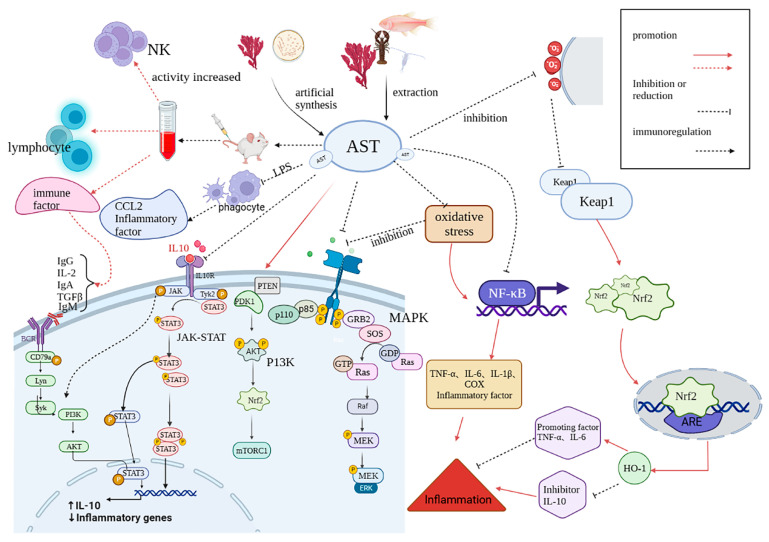
The anti-inflammatory and immunomodulatory mechanisms of AST.

The varying antioxidant and anti-inflammatory properties of AST forms produced via chemical synthesis exhibit distinctions. They are categorized into three forms: non-esterified, monoesterified, and diesterified [48]. The solubility and dispersibility of different forms of AST are different, which affects the oxidation activity and anti-inflammatory activity. In addition, AST has the potential to prevent excessive bone loss, dermatitis, neuroinflammation, and fungal keratitis and reduce inflammatory pain. The anti-inflammatory mechanisms of AST are shown in Figure 5.

### 3.5. Immunoregulation

One of the important components of immunity is to reduce inflammation. The two are interrelated and influence each other. Inflammation is characterized by a cytokine storm resulting from elevated levels of cytokines like IL-6. AST can suppress pro-inflammatory cytokines and chemokine C C motif ligand 2 (CCL2) in LPS-stimulated macrophages by enhancing lymphocyte and natural killer cell activity [50], thereby boosting the immune response [146]. AST’s distinctive molecular composition facilitates its passage through the blood–brain barrier, a key factor in the prevention and management of neurological disorders [147,148]. In related research, Zhu et al. [51] supplemented hen diets with varying levels of AST-rich *phaffia rhodozyma* (PR) and compared the serum immunoglobulin levels in the test hens. The group with elevated AST levels exhibited a marked rise in serum IgG levels (*p* < 0.05), implying that a balanced AST dosage can bolster the immune system in laying hens. Zhang and colleagues [52] dosed cyclophosphamide-induced immunodeficient mice with varying amounts of AST (30, 60, and 120 mg/kg body weight). It was found that the high-dose groups could effectively prevent intestinal mucosal damage, including reducing the level of oxidative stress (like MDA, GSH, and GSH-Px). They kept the gut’s structure solid, nurtured helpful goblet cells to multiply and produce more mucus, and dialed down the production of Paneth cells and their antimicrobial peptides (like regenerating family member gamma (Reg-3γ) and lysozyme). Plus, they boosted the production of IgA, which is key for immunity. Enhancing the primary gut microbiota, including total bacteria, lactic acid bacteria, and Enterobacteriaceae, along with their metabolites, shows that AST enhances immunity by increasing serum immune factors and their activity while supporting intestinal health in immunodeficient subjects.

AST can delay immune decline and increase the activity of immune factors. Immunity decline is intricately tied to aging, marked primarily by a decline in both innate and adaptive immune systems. Research shows that oxidative stress and immune organ shrinkage accelerate immune cell deterioration, hastening immune decline. AST can slow down aging by reducing oxidative stress and bolstering the immune system, revealing its potential to heal liver tissue injuries. AST’s anti-aging effects are partly linked to the Nrf2/Keap1 and NF-κB signaling pathways [53]. AST suppresses the NF-κB pathway, lowering inflammatory mediators. The Nrf2/Keap1 pathway modulates inflammation by controlling cytokine expression.

Fan et al. [149] treated rodents with daily intakes of 4.2, 8.35, and 16.70 mg/kg of body mass over a 30-day period. Measurements included the spleen and thymus indices, as well as spleen lymphocyte transformation activity and additional data. AST administered at moderate to high doses markedly boosted immune function across multiple measures, including spleen lymphocyte proliferation and transformation, antibody-producing cell activity, serum hemolysin concentrations, and macrophage efficiency (as reflected in the carbon clearance phagocytic index)—all showing statistically significant improvements over baseline control levels. A high dose notably enhanced both delayed allergic reactions and NK cell activity. AST enhances NK cell immune factors such as IgA and IgG, boosts lymphocyte activity and transformation, improves reproductive capacity, and protects immune organs, thereby strengthening the body’s immunity. The immune regulation mechanisms of AST are shown in Figure 5.

### 3.6. Skin Care

Ultraviolet irradiation can lead to changes in skin structure, pigmentation, loss of skin elasticity, dry skin, wrinkles, and delayed wound healing, thereby accelerating skin aging [150]. AST can maintain skin health and solve skin damage by anti-inflammatory and anti-oxidation effects, regulating apoptosis, regulating aquaporin, skin flap repair, and reducing pigmentation.

Atopic dermatitis (AD) is a persistent inflammatory skin disorder, where thymus- and activation-regulated chemokine (TARC) and MDC are key players in its development. Hà et al. [54] evaluated the immunomodulatory effects of *Pyropia yezoensis* extracts (PYE) against IFNγ- and TNF-α-induced responses in a HaCaT keratinocyte culture model. The study showed a reduction in the production of Th2 chemokines with PYE, including TARC and MDC, in HaCaT cells. These effects are achieved by blocking IκB-α breakdown, blocking the phosphorylation of ERK, JNK, and p38, and ultimately preventing the nuclear translocation of NF-κB/p65. Additionally, 0.08 μg/mL AST and 0.05 μg/mL lutein were obtained from a 10 mg/mL PYE solution. The reaction effect of these two substances is similar to that of PYE, indicating that AST and lutein are the main substances in PYE. Ultraviolet radiation enhances ROS and inflammatory intermediate production while directly damaging skin cell DNA. Chung et al. [55] pretreated normal human epidermal keratinocytes (NHEKs) with 20 μM AST for 24 h before subjecting them to ultraviolet B (UVB) irradiation. Cell viability and apoptosis-related protein expression were assessed after 24 h. The research revealed that administering AST to NHEKs prior to UVB exposure effectively counteracts oxidative stress and programmed cell death. This protective mechanism occurs through the down-regulation of pro-apoptotic markers (Bax, Caspase3, and poly ADP-ribose polymerase (PARP)) coupled with a reduction in Bcl-2 expression, ultimately shielding skin cells from UVB damage. AST exhibits dual roles in apoptosis regulation, both inhibiting and promoting the process to protect the skin. It down-regulates anti-apoptotic proteins (Bcl-2, p-Bad, and survivin) while up-regulating pro-apoptotic proteins (Bax, Bad, and PARP), thereby inhibiting melanoma cell proliferation and demonstrating potential for treating skin tumors [127]. Aquaporin-3 (AQP3) is crucial for preserving skin hydration and functionality. Ikarashi et al. demonstrated that AST elevates mRNA and protein expression levels of AQP3 and enhances AQP3’s glycerol permeability and that both oral and topical applications amplify water effects [23]. Gürsoy et al. [56] discovered that administering AST over 4 mg/kg enhances flap viability and stimulates vascular formation, qualifying it as a supportive agent in skin defect repairs. Honda and colleagues [57] examined how different ratios of AST isomers affected UV-induced skin damage in guinea pigs when administered orally. The animals were divided into two dietary groups for a three-week period: one group received a diet high in all-E isomer AST (E-AST-D), containing only 3.2% Z-isomers, while the other was given a Z-isomer-rich diet (Z-AST-D) with 84.4% Z-isomers. Experimental results indicated that both diets mitigated ultraviolet-induced skin damage, with Z-AST-D demonstrating superior efficacy. This was evidenced by its enhanced ultraviolet shielding capability and more significant reduction in skin pigmentation, including melanin and erythema values, due to ultraviolet exposure.

### 3.7. Antidiabetic

Type 2 diabetes mellitus, characterized by insulin insensitivity and a comparative insulin shortage, is the most common chronic metabolic disorder affecting global health [58]. Oxidative stress plays a pivotal role in diabetes’ onset, progression, and long-term complications. Elevated blood sugar levels trigger excessive ROS, which wreak havoc across multiple biological systems in diabetic patients. This oxidative stress damages pancreatic β cells’ ability to produce insulin, weakens target tissues’ responsiveness to insulin signaling, and compromises vascular endothelial function, regardless of whether patients have type 1 or type 2 diabetes. The cascade of cellular dysfunction ultimately creates a vicious cycle that exacerbates diabetic complications. AST alleviates cellular oxidative stress and reduces inflammation through ROS elimination and the suppression of lipid oxidation [151], thus mitigating diabetes-related oxidative stress and aiding treatment. Chen et al. [30] employed pregnant C57BL/KsJ db/+mice to model gestational diabetes mellitus (GDM) genetically. The research results indicate that AST may become a key approach for treating diabetes and gestational diabetes symptoms. AST could effectively alleviate the symptoms of GDM by activating antioxidant enzymes (SOD, GPX, CAT), the Nrf2/HO-1 signaling pathway, and improving glucose intolerance and β-cell dysfunction. Zhuge et al. [58] developed a streptozotocin (STZ)-induced diabetic rat model, revealing that diabetic rats exhibited elevated blood glucose and total cholesterol (TC) levels compared to normal mice, alongside reduced expression of insulin sensitivity-related genes, including adiponectin, adiponectin receptor 1 (AdipoR1), adiponectin receptor 2 (AdipoR2), and PPARγ. AST exhibits a substantial reduction in STZ-induced diabetic rat model effects in a dose-responsive fashion. High plasma homocysteine (Hcy) levels can significantly harm vascular endothelial cells. AST reduces Hcy-related endothelial impairment by inhibiting VEGF-VEGF receptor 2 (VEGFR2)-focal adhesion kinase (FAK) pathway activation [59]. AST could aid in preventing diabetes-related issues like retinopathy, nephropathy, neuropathy, and cardiovascular disease [151].

### 3.8. Neuroprotection

AST efficiently crosses the blood–brain barrier and significantly influences cell signal regulation. It safeguards neurons by reducing oxidative stress, suppressing inflammation, and modulating nerve-signaling enzyme expression.

AST can produce neuroprotective effects through antioxidant effects. Excessive ROS generation, indicative of oxidative stress, can harm mitochondria through various mechanisms, possibly leading to neurodegenerative conditions [152]. In an experimental mouse model of traumatic brain injury, treatment with AST demonstrated a marked, dose-dependent increase in the expression of silent information regulator 1 (SIRT1), Nrf2, and peroxiredoxin 2 within primary cortical neurons subjected to oxidative stress from H_2_O_2_. Concurrently, it suppressed the activation of apoptosis signal-regulating kinase-1 (p-ASK1) and phosphorylated p38, indicating a neuroprotective effect. Among them, peroxiredoxin 2 can remove various peroxides including H_2_O_2_ through certain redox reactions on cysteine residues, thereby achieving neuroprotective effects [24]. Aneurysmal subarachnoid hemorrhage (SAH) is associated with significant mortality and morbidity. Early brain injury (EBI) worsens SAH outcomes, primarily due to oxidative stress post-SAH. Experiments indicate that AST treatment for SAH significantly decreases MDA levels in brain tissue, enhances SOD and GSH expression, and restores endogenous antioxidant levels, thereby providing neuroprotection [61]. Zhang’s [62] OGD model study revealed AST’s activation of the PI3K/Akt/glycogen synthase kinase-3β (GSK3β)/Nrf2 pathway. This activation up-regulates HO-1, Nrf2, p-Akt/Akt, and p-GSK3β/GSK3β expression, significantly reduces ROS levels, inhibits oxidative cell damage, decreases neuronal apoptosis under OGD conditions, and provides neuroprotection.

AST can indirectly achieve neuroprotective effects through its own anti-inflammatory activity. PM2.5 refers to particles in the air with a diameter less than 2.5 μm. It is a key component in air pollution. It can bypass the defense mechanisms of the brain and may play a role in triggering and exacerbating conditions such as Alzheimer’s disease, Parkinson’s disease, and various forms of dementia [153]. Studies show that exposure to PM2.5 particles triggers increased expression of iNOS and HO-1 in BV-1 microglial cells, shifting them toward pro-inflammatory M2 and disease-associated microglia (DAM) states while dampening their anti-inflammatory functions. This imbalance ultimately contributes to neurotoxic effects. AST reduces pro-inflammatory M1 and DAM polarization through NF-κB and Nrf2 signaling, alleviates neurotoxicity, and achieves neuroprotection [63].

AST exerts neuroprotective effects by regulating neurotransmitter activity. Doxorubicin (DOX), an anticancer agent, exhibits pronounced neurotoxic effects, leading to cognitive deterioration. The process boosts lipid and protein oxidation, reduces GSH levels, and alters antioxidant enzyme equilibrium. AST mitigates DOX-induced neurotoxicity by decreasing acetylcholinesterase (AChE) activity, lowering TNF-α, prostaglandin E2 (PGE2), and cyclooxygenase-2 (COX-2) levels, and inhibiting cytochrome C expression [64]. Moreover, pretreatment with AST has been shown to lower ROS generation and minimize lipid peroxidation in the affected brain hemisphere of rats subjected to middle cerebral artery occlusion (MCAO). This protective effect not only reduces the extent of cerebral damage but also promotes better recovery of motor abilities [154]. In their study, Wang and colleagues [60] failed to take advantage of the MCAO rat model to illustrate how boosting cyclic adenosine monophosphate (cAMP) concentrations within the brain tissue sets off the cAMP/cAMP-response element binding protein (CREB)/protein kinase A (PKA) signaling cascade, which in turn fosters the regeneration of axons in the cerebral cortex.

### 3.9. Others

Beyond these primary biological functions, AST also has antibacterial, antiviral, antidepressant, and other biological activities. In their study on AGS cells infected with Helicobacter pylori, Li and colleagues [155] demonstrated that AST can curb the expression of MMPs, cell invasion, and migration by dampening the PI3K/AKT/mTOR/NF-κB signaling pathway induced by the bacterium, effectively saving the *Helicobacter pylori*-induced gastric cancer cells from being invaded. Moreover, AST boasts a lower minimum inhibitory concentration (MIC) against Staphylococcus aureus than novobiocin. Treatment with AST leads to a marked boost in superoxide anion levels and a decrease in GSH concentrations while increasing the ADP/ATP ratio compared to DMSO-treated cells. Additionally, AST forms a more stable and relevant complex with topoisomerase IV ParC and ParE than reference antibiotics. This suggests a strong link between AST’s antibacterial prowess and oxidative stress [156]. A recent study has revealed that AST can prevent HPV16-L1 from triggering sperm membrane rearrangement specifically by lessening the displacement of head CTB and Lyn, reducing Tyr phosphorylation (Tyr-P) levels, and diminishing the percentage of acrosome reaction cells (ARC), ultimately preserving sperm function by cutting down the amount of HPV16-L1 capsid proteins that bind to the human sperm membrane [157]. Research by Li et al. [158] also highlighted that AST effectively suppresses *herpes simplex virus*-1 (HSV) infection and prevents oxidative stress-induced stimulator of interferon genes (STING) carbonylation. Furthermore, it facilitates the translocation of STING to the Golgi apparatus and promotes its oligomerization, ultimately triggering STING-dependent host defense mechanisms. This demonstrates AST’s potent antiviral properties. Recent studies have revealed that trans-AST exhibits promising antidepressant properties by modulating serotonergic activity. Researchers observed that this compound suppresses indoleamine 2,3-dioxygenase (IDO) expression in the hippocampus and spinal cord while regulating key metabolic ratios involving kynurenine/tryptophan and serotonin pathways. Additionally, its anti-inflammatory effects, achieved through the antagonism of pro-inflammatory cytokines including IL-1β, IL-6, and TNF-α, contribute to alleviating neuropathic pain behaviors in controlled cortical impact (CCI) model mice. These improvements were demonstrated by reduced mechanical allodynia, thermal hyperalgesia, and depressive-like behaviors, as evidenced by shorter immobility times during forced swim and tail suspension tests. The findings collectively suggest AST’s potential as a multifaceted therapeutic agent for both pain management and mood regulation [159]. Notably, AST has also demonstrated beneficial effects on exercise performance. In an experimental study, AST significantly improved insulin resistance and impaired glucose tolerance in high-fat-diet mice by regulating AMPK activation in muscles. It could stimulate mitochondrial biogenesis in muscles and enhance exercise tolerance and exercise-induced fatty acid metabolism. Besides, it exerted anti-inflammatory effects through its antioxidant activity in adipose tissue [160]. A systematic review and meta-analysis showed that AST supplementation, particularly at high doses (≥20 mg/day) and long durations (>12 weeks), significantly enhances aerobic exercise performance and physical function, promotes fat oxidation, alleviates subjective fatigue, and exhibits a mild improvement trend in cognitive accuracy, with more pronounced effects in aerobic exercise contexts [161].

## 4. Conclusions, Challenges, Opportunities and Future Perspective

AST is a type of carotenoid that is widely present in nature. Through continuous theoretical discussions and experimental research by numerous scholars at home and abroad, we have now clarified the molecular structure and biosynthesis pathway of AST. Based on traditional extraction methods, we have also proposed several new green extraction processes for AST, such as SFE, UAE, PEF, HPP, etc. Additionally, we summarize AST’s various biological activities, including antioxidant and anti-tumor effects. Due to its outstanding biological activity, AST shows great application potential in the fields of medicine and health, cosmetics, food industry, and aquaculture. In the medical field, it can be used for the prevention of chronic diseases, as an auxiliary treatment for cancer, and for the development of new drugs [37,162,163]. In the cosmetics industry, it can serve as a key component for anti-aging and skin repair [164,165]. In the food industry, it can also serve as a natural coloring agent, an antioxidant preservative, etc. [70,166]. However, although AST has broad application prospects, future research on AST still faces many challenges. We believe that the following aspects are the current frontier problems that urgently need to be addressed in AST research: ① The production methods and stability of AST (including, but not limited to, the low biosynthesis rate and long cycle of natural AST; the cumbersome steps and unstable products of chemically synthesized AST). ② The existing research models are monotonous, with simplistic mechanisms of action. In addition, the specific effects of different isomers have not been clearly identified, which has led to the fact that the current research cannot provide more of a theoretical basis for the true extrapolation of AST to humans and clinical applications. ③ We still need to accelerate the construction of a safety assessment system for synthetic AST and deeply explore the potential biocompatibility risks and long-term exposure effects of new delivery systems for AST (such as nanocarriers, liposomes) in different application scenarios (such as food, medicine, cosmetics, etc.).

It is well known that the production of AST currently faces the dual challenges of low efficiency in natural biosynthesis and complex chemical synthesis processes, which greatly restricts the commercial production of AST. Microalgae and other natural organisms are the best sources of natural AST. This type of AST, due to its high purity and all-trans configuration advantages, is widely used in the fields of pharmaceuticals, aquaculture, health foods, and cosmetics [167]. However, due to the high production cost of microalgae, the accumulation efficiency is low, and during the extraction process, AST is exposed to high temperatures, oxygen, light, and extreme pH environments for a long time, resulting in chemical instability, which will affect and damage its practical application. In addition, the cyst-like rigid structure inherent in microalgae will also form a “physical barrier”, reducing the extraction efficiency [92]. Although various modern green extraction processes have been proposed, which have partially addressed the influence of external conditions and the properties of microalgae on the extraction efficiency during the extraction of AST, due to the low accumulation efficiency of AST by microalgae, the yield of AST has still not been significantly increased. Moreover, these methods are only at the theoretical research stage and have not been widely applied and verified in the food industry. Adequate biomass production is the primary goal in a sustainable biorefining process. From the current technical bottlenecks and industrial demands, the key to breaking through the limit of natural AST yield still needs to be traced back to the biological synthesis source. In recent years, CRISPR/Cas9 gene editing technology has provided a new perspective for addressing the low efficiency and high cost of natural AST accumulation. Kneip et al. [168] used CRISPR/Cas9 technology to repeatedly overexpress the chlamydomonas reinhardtii β–carotene ketolase (CrBKT), pantoea agglomerans CrtB (PacrtB), and Chlamydomonas reinhardtii β–carotene hydroxylase (CrCHYB) genes in microalgae, resulting in a 2.3-fold increase in AST accumulation in the mutant ΔLCYE#3 (reaching 1.8 milligrams per liter), which was higher than that of the parental strain UVM4. This demonstrates the potential of gene editing technology in increasing the yield of natural AST. However, at present, this technology is still in its infancy in terms of enhancing the accumulation of AST in natural products. Further in-depth exploration is needed, and the proposed green extraction process for AST still requires further optimization. It awaits verification through large-scale production in the food industry. Compared to the biological synthesis of AST, although the chemical synthesis of AST has achieved large-scale production, most of its reaction processes rely on toxic organic solvents, which can easily cause heavy metal residues and isomer impurity pollution. Although this chemical synthesis of AST has a lower cost, due to its potential toxicity, it has been explicitly prohibited by the FDA from entering the food and health supplement markets. Seeking a cost-effective, safe, efficient, and stable chemical synthesis route remains an important research direction for the large-scale production of AST.

Research has demonstrated that AST possesses a wide range of biological activities, covering areas such as antioxidant, anti-inflammatory, and anti-apoptotic effects. However, the existing studies still have significant limitations: Firstly, most mechanism studies rely on in vitro cell models or animal experiments, and the types of research models are relatively limited. Secondly, there is a lack of large-scale human clinical trial data to support the results, and the concentration of AST used in in vitro experiments (at the μM level) is much higher than the plasma concentration that can be achieved in the human body (typically <1 μM), resulting in a significant gap in result transformation. Thirdly, existing studies mostly focus on a single signaling pathway, and the interpretation of the mechanism is fragmented, lacking systematic research on the synergistic effect of AST with other biomolecules (such as fatty acids, proteins) and the cross-regulatory mechanism of multiple pathways. Fourthly, the specific effects of AST isomers have not been clarified, and the differences in biological activity and metabolic mechanisms of different configurations still need to be deeply explored. Fifthly, the complex metabolic process of AST in the body (such as intestinal microbial metabolism) and the biological activity of its metabolites have not been fully revealed. Future research can be conducted based on multi-omics technologies, organoid model construction, and clinical cohort studies to systematically investigate the biological activity network of AST.

Current research indicates that natural AST does not exhibit any toxicity at any dosage or within any time frame. There are no records of serious adverse events for short-term daily doses (up to 100 milligrams) and long-term daily doses averaging 8 to 12 milligrams [169]. However, there are very few safety studies on synthetic AST conducted in humans or animals. Therefore, it is necessary to assess the safety of synthetic AST, determine its safety parameters, and provide a theoretical basis for the future application of synthetic AST. In addition, since there are unsaturated bonds in the structure of AST, AST is susceptible to heat, oxygen, light, and moisture. To better apply it in production, researchers have proposed methods such as nano encapsulation, microencapsulation, and emulsification to prevent the rapid loss of antioxidant performance. However, these insoluble and non-degradable nanoparticles may accumulate in target organs, and the long-term risks of nanomaterials deserve attention. Besides, is there any possibility for us to develop more convenient and efficient protection methods to enable AST to be applied in more fields? Further exploration is needed.

In conclusion, AST plays a significant role in protecting human health, enhancing food taste and safety, and meeting various life-related needs. This review may contribute to promoting the safe development and application of AST in multiple fields such as food, medicine, cosmetics, and aquaculture, accelerating its transformation from laboratory research to functional food ingredients, natural drug precursors, and green agricultural additives. In the future, it is necessary to achieve revolutionary breakthroughs in the synthesis technology of AST and its safety assessment through continuous integration of multiple disciplines.

## Figures and Tables

**Table 1:** Mechanisms of AST biological activity (↑ increase, ↓ decrease).

Bioactivity	Models	Mechanisms	References
Antioxidation	Mice aging model	↓ MDA, NO, AOPP ↑ SOD, GSH, CAT	[26]
	Mouse model of chronic obstructive pulmonary disease	↓ MDA ↑ SOD, GSH, HO-1, T-AOC, Nrf2	[27]
	Memory loss mouse model	↓ TNF-α, IL-1β, IL-6, ROS, NO	[28]
	Aging cock model	↓ MDA, -OH, O_2_^−^ ↑ SOD, GSH-Px, CAT, T-AOC, HO-1, ERK, p38, JNK1/2/3, Nrf2	[29]
	Genetic gestational diabetes mellitus model	↓ Glucose intolerance ↑ SOD, GSH-Px, CAT, Nrf2, HO-1	[30]
Anti-cancer	Obese mouse colon cancer model	↓ Cell proliferation, oxidative stress, inflammatory response, NF-κB	[31]
	Human gastric cancer cell line AGS cells	↑ NQO1, ROS, p-RIP1	[32]
	Rat model of esophageal cancer	↓ MDA ↑ T-AOC, SOD, PPARγ, Bax/Bcl-2, Caspase3	[33]
	Breast cancer cell line SKBR3 cells	↓ mutp53, Cleavage of PARP-1 fragment, Bcl2, ROS ↑ G0/G1 cycle arrest, Bax, ERK1/2, p-JNK, p-p38, SOD Regulating bridge protein	[34]
	Prostate cancer du145 cell	↓ Cell migration and proliferation, STAT3 ↑ Apoptosis	[35]
		↓ p-Akt/Akt, NF-κB, STAT3, Wnt	[36]
	Colorectal cancer cells CT26 and HCT116	↓ EMT, MMP2, ZEB1 ↑ miR-29a-3p, miR-200a	[37]
	Melanoma cell A375 and A2058 models	↓ MMP1/2/9 ↑ caspase3, caspase7	[38]
	Astrocytic glioma cell line U251-MG	↓ p53 ↑ Cdk2, p-Cdk2/3	[39]
Eye-protection	Retinal ischemia/reperfusion model	↓ ERG, ROS	[40]
	Type I diabetic rat model	↓ AGE, MDA, Lipid hydrogen peroxide ↑ CAT, SOD, GSH	[41]
	DED rat model	↓ SPK lesions	[42]
	DED mouse model and HCECs cells	↓ HMGB1, TNF-α, IL-1β ↑ p-Akt	[43]
	ARPE-19 cells and HIR mouse model	↓ HIF1α, XBP1, VEGF, Cell permeability Protect ZO-1	[44]
	661w cells	↓ ROS, Nitrotyrosine, 8-OHdG, Acrolein, Mitochondrial damage ↑ Bcl-2/Bax, PI3K/Akt, HO-1, NQO1	[45]
Anti-inflammatory	MCAO rats’ model	↑ SOD, HO-1, NQO1 ↓ O_2_^−^, MDA	[46]
	Mouse primary peritoneal macrophage model	↓ IκB-α, ERK1/2, P38, JNK	[47]
	Mouse macrophage Raw 264.7 cell model	↓ TNF-α, IL-1β, IL-6	[48]
	Mouse macrophage Raw 264.7 model	↓ NF-κB, NFATC1	[49]
Immunoregulation	NASH mouse model	↓ Proinflammatory cytokine, CCL2	[50]
	Roman Brown laying hens	↓ MDA ↑ GSH-Px, SOD, IgG	[51]
	C57BL/6 mice	↑ IgA ↓ Reg-3γ, Lysozyme	[52]
	Male Sprague Dawley rats	↓ IL-2, IgM, IgG, IL-1β, IL-6, IκBα, p65 ↑ Nrf2, Keap1	[53]
Skin care	HaCaT	↓ IκB-α, ERK, JNK, p38, p65	[54]
	NHEKs	↓ Bax, PARP, ROS, Caspase3 ↑ Bcl-2	[55]
	Episkin 3D human skin model	↑ AQP3	[23]
	Sprague Dawley rats	↑ Flap survival rate, Angiogenesis	[56]
	UV light-induced guinea pig model	↓ Pigmentation, decreased elasticity, transcutaneous water loss	[57]
Antidiabetic	Genetic gestational diabetes mellitus model	↓ Glucose intolerance ↑ SOD, GSH-Px, CAT, Nrf2, HO-1	[30]
	Streptozotocin diabetic rat model	↑ Adiponecti, AdipoR1, AdipoR2, PPARγ ↓ TC	[58]
	HuVecs	↓ VEGF-VEGFR2-FAK signaling pathway	[59]
Neuroprotection	Mouse model of traumatic brain injury	↓ p-ASK1, p-p38 ↑ SIRT1, Nrf2, Prxs	[24]
	MCAO rats’ model	↑ cAMP, CREB, PKA	[60]
	db/db mice	↓ MDA ↑ GSH, SOD	[61]
	Human neuroblastoma SH-SY5Y cells	↓ HO-1, Nrf2, p-Akt/Akt, p-GSK3β/GSK3β	[62]
	BV-2 microglial cells	↓ iNOS, NF-κB, ↑ Nrf2, HO-1	[63]
	Male albino rats	↓ AChE, TNF-α, PGE2, COX-2, Cytochrome c	[64]

8-OHdG, 8-hydroxy-2 deoxyguanosine; AChE, acetylcholinesterase; Akt, protein kinase B; AOPP, advanced oxidative protein product; AQP3, aquaporin-3; ASK1, apoptosis signal regulating kinase-1; Bax, BCL2 associated X protein; cAMP, cyclic adenosine monophosphate; CAT, catalase; CCL2, C C motif ligand 2; Cdk, cyclin-dependent kinases; COX-2, cyclooxygenase-2; CREB, cAMP-response element binding protein; EMT, epithelial–mesenchymal transition; ERG, electroretinogram; ERK, extracellular signal-regulated kinase; FAK, focal adhesion kinase; GSH, glutathione; GSH-Px, glutathione peroxidase; GSK3β, glycogen synthase kinase-3β; HIF1α, hypoxia-inducible factor 1α; HMGB1, high-mobility group box 1; HO-1, heme oxygenase 1; IκB-α, NF-κB inhibitor alpha; IL, interleukin; iNOS, inducible nitric oxide synthase; JNK, c-Jun N-terminal kinase; Keap1, Kelch-like ECH-associated protein 1; MDA, malondialdehyde; MMP, matrix metalloproteinases; NF-κB, nuclear factor kappa-B; NFATC1, nuclear factor of activated T cells 1; NQO1, NAD (P) H quinone dehydrogenase 1; Nrf2, nuclear factor (erythroid-derived 2)-like 2; PARP-1, poly ADP-ribose polymerase-1; PGE2, prostaglandin E2; PKA, protein kinase A; PPARγ, peroxisome proliferator-activated receptor γ; Prxs, peroxiredoxin; Reg-3γ, regenerating family member gamma; RIP1, receptor-interacting protein 1; ROS, reactive oxygen species; SIRT1, silent information regulator 1; SOD, superoxide dismutase; SPK, superficial punctate keratopathy; STAT3, signal transducer and activator of transcription 3; T-AOC, total antioxidant capacity; TC, total cholesterol; TNF-α, tumor necrosis factor-α; VEGF, vascular endothelial growth factor; VEGFR2, VEGF receptor 2; Wnt, wingless/integrated; XBP1, X-box binding protein 1; ZEB1, zinc finger e-box binding homeobox 1; ZO-1, zonula occludens-1.

**Table 2:** Comparison of the advantages and disadvantages of different AST production methods.

Production Methods	Classification	Advantages and Disadvantages	References
Biological accumulation	Direct extraction of waste from crustaceans	Low output and high costs.	[65]
Traditional extraction	Solvent extraction	The overall processing conditions are very strict, resulting in poor quality, yield, and stability of AST; high energy consumption and multiple separation steps.	[71,72]
	Oil extraction		
Chemical synthesis	Total synthesis method	The cost is relatively low, with a market application rate of 90%. It is mainly used in aquaculture and can produce a mixture of AST stereoisomers (L: racemic: R in a ratio of 1: 2: 1); there are unknown components and potential risks.	[70]
	Semi-synthesis		
Modern green technology	SFE-CO_2_	Low viscosity, high diffusivity, and high density, enhancing the penetration of the biomass structure and the dissolution of target compounds; reducing extraction time and solvent usage.	[73]
	UAE	The oxidation of lipids was triggered and intensified, resulting in significant increases in PV and TBARS. Among them, the presence of tannic acid (0.1%) resulted in the highest yield.	[74]
	PEF	Increases the extraction rate of lipid and carotenoid components in the shrimp’s head and thorax; has an inhibitory effect on enzymes; increases the content of bioactive substances in the oil.	[75]
	HPP	High quality; green and pollution-free; short time consumption.	[76]
Biosynthesis	*H. pluvialis*	Maintains high photosynthetic activity and promotes the biosynthesis of AST; microalgae grow slowly and the production process is relatively long, making them prone to contamination during the production stage.	[67]
	*Coelastrum* sp. HA-1	Under the original conditions, the esterification rate of AST molecules was low, and the accumulation of AST was lower than that of *H. pluvialis*. When LA and ethanol were added, the content of AST esters and TA increased exponentially.	[77]
	*X. dendrorhous*	Providing yeast and AST with abundant carbon sources, nitrogen sources, minerals, and arabinose can significantly promote and increase the production and yield of AST.	[70]
	*E. coli* CAR026	It cannot accumulate AST by itself, but it can enhance the tolerance and production of AST.	[78]

AST, astaxanthin; *E. coli*, *Escherichia coli*; *H. pluvialis*, *Haematococcus pluvialis*; HPP, high-pressure treatment; LA, linoleic acid; PEF, pulsed electric field; PV, peroxide value; SFE-CO_2_, supercritical carbon dioxide; TA, total AST; TBARS, thiobarbituric acid active substance; UAE, ultrasonic-assisted extraction; *X. dendrorhous*, *Xanthophyllomyces dendrorhous*.

**Table 3:** The extraction methods and sources of AST.

Production Mechanisms	Methods	Sources or Strains	Yield	References
Active synthesis	Microbial Biosynthesis	Mutant strain named as M3 of *H. pluvialis*	The accumulation of fatty acids and AST was higher than that of wild strains.	[82]
		*H. pluvialis*	The contents of AST and lipids were increased by 77.57% and 23.39%, respectively.	[83]
		*Coelastrum* sp. HA-1	The contents of AST esters and TA were 3.82 times and 2.18 times (treated with LA) or 2.42 times and 1.61 times (treated with ethanol) those of the control group, respectively.	[77]
		*X. dendrorhous*	Ponkan peel extract used alone (40 g/L): 0.92 mg/L; Ponkan peel extract was added to Synthetic Dropout medium: 1.22 mg/L; Ponkan peel extract was added to YM medium: 2.05 mg/L.	[70]
		*E. coli* CAR026 (Coordinate the expression of CrtW and CrtZ, increase the copy number of crtY, and regulate groES-groEL)	1.18 g/L	[85]
Passive accumulation	SFE-CO_2_ (300 bar, 60 °C and 6 mL/min)	Shrimp residue lactic acid fermentation broth	0.6353 μg/g	[87]
	SPD (TFA obtained from crude viscera oil as solvent) (160 °C, 0.002 mbar)	By-products of *Parapenaeus longirostris*	114.80 ± 1.23 µg/mL	[96]
	Combination of MW pretreatment and SFE (0–30 min, 200–500 bar, 40–60 °C), ethanol content (8–13 wt%)	Brown crab (*Cancer pagurus*) shell waste	1023 μg/g	[97]
	UAE (ultrasonic amplitude: 23.6%, 13.9 min, 26.3 °C)	*Penaeus semisulcatus* shell	It accounted for 51.5% of the extract.	[74]
	UAE (Preheat and add 0.1% tannic acid) (ultrasound amplitude: 80%, 25 min)	Pacific white shrimp (*Litopenaeus vannamei*)	Lipid: 133–141 mg/g sample	[100]
	UAE (ultrasound amplitude: 80%, 25 min) plus PEF pretreatment	Pacific white shrimp (*Litopenaeus vannamei*)	Lipid: 303.4 mg/g solids	[99]
	HPP (acetone and methanol (7: 3, *v*/*v*), 210 MPa, 10 min)	*Penaeus monodon*	59.9744 µg/gdw total carotenoid: 68.26 µg/ml	[76]
	Enzyme-assisted extraction (20 units of *P. segnis* digestive alkaline proteases/g of blue crab shells for 60 min at 50 °C and pH 8.0.) combined with impregnation (MAC by using the binary organic system HxIPA (50/50, *v*/*v*), 120 h, solvent/raw material ratio of 4/1 (*v*/*w*))	Blue crab (*Portunus segnis*)	5045 µg/g	[102]
	microbiological degradation (the culture media-conditions is pH 7.0, monosodium glutamate 3% (*w*/*v*), glucose (1% *w*/*v*) and 30 °C)	SSW	2.16 U/mL	[103]

AST, astaxanthin; CrtW, β-carotene ketolase; CrtY, lycopene cyclase; CrtZ, β-carotene hydroxylase; *E. coli*, *Escherichia coli*; *H. pluvialis*, *Haematococcus pluvialis*; HPP, high-pressure processing; LA, linoleic acid; MAC, maceration extraction technique; MW, microwave; PEF, pulsed electric field; SPD, short-path distillation; SFE, supercritical fluid extraction; SFE-CO_2_, supercritical carbon dioxide extraction; SSW, shrimp shell waste; TA, total AST; TFA, total fatty acids ethyl esters; UAE, ultrasonic-assisted extraction; *X. dendrorhous*, *Xanthophyllomyces dendrorhous*.

## Data Availability

No new data were created or analyzed in this study. Data sharing is not applicable to this article.

## Abbreviations

8-OHdG, 8-hydroxy-2-deoxyguanosine; ABTS, 2, 2′-azino-bis(3-ethylbenzothiazoline-6-sulfonic acid); AChE, acetylcholinesterase; AD, atopic dermatitis; AdipoR, adiponectin receptor; AGE, advanced glycation end products; Akt, protein kinase B; AOM, azoxymethane; AQP3, Aquaporin-3; ARC, acrosome reaction cells; ASK1, apoptosis signal regulating kinase-1; AST, astaxanthin; Bax, BCL2-Associated X Protein; BRB, blood–retinal barrier; cAMP, cyclic adenosine monophosphate; CAT, catalase; Caspase3, cysteinyl aspartate specific proteinase 3; CCI, controlled cortical impact; CCL2, C-C motif chemokine ligand 2; Cdk, cyclin-dependent kinase; cAMP, cyclic adenosine monophosphate; CIS, cisplatin; COX-2, cyclooxygenase-2; CrBKT, chlamydomonas reinhardtii β–carotene ketolase; CrCHYB, Chlamydomonas reinhardtii β–carotene hydroxylase; CREB, cAMP-response element binding protein; CrtW, β-carotene ketolase; CrtY, lycopene cyclase; CrtZ, β-carotene hydroxylase; CSC, cancer stem cell; DAM, disease-associated microglia; DC, diabetic cataract; DCW, dry cell weight; DED, dry eye disease; DMAPP, dimethylallyl pyrophosphate; DOX, Doxorubicin; DPPH, 1,1-diphenyl-2-picrylhydrazyl; DR, diabetic retinopathy; E-AST-D, E isomer AST; EBI, early brain injury; *E. coli*, *Escherichia coli*; EMT, epithelial-mesenchymal transition; ERG, electroretinogram; ERK, Extracellular signal-regulated kinase; FAK, focal adhesion kinase; FBP, fructose-1,6-bisphosphatase; GDM, gestational diabetes mellitus; GGPP, geranylgeranyl diphosphate; GSH, glutathione; GSH-Px, glutathione peroxidase; GSK3β, glycogen synthase kinase-3β; GSTs, glutathione S-transferases; HCECs, human corneal epithelial cells; Hcy, High-plasma homocysteine; HIF1α, hypoxia-inducible factor 1α; HIR, hyperoxia-induced retinopathy; HMGB1, high-mobility group box 1; HO-1, heme oxygenase-1; HPP, high-pressure treatment; HSV, herpes simplex virus-1; *H. pluvialis*, *Haematococcus pluvialis*; IDO, indoleamine 2,3-dioxygenase; IL, Interleukin; IPP, isopentenyl pyrophosphate; iNOS, Inducible Nitric Oxide Synthase; IP, intraperitoneal; IV, intravitreal; JNK, c-Jun N-terminal kinase; Keap1, Kelch-like ECH-associated protein 1; LA, linoleic acid; LDH, lactate dehydrogenase; LED, light-emitting diode; LPS, lipopolysaccharide; LTP, low-temperature plasma; MAPK, mitogen-activated protein kinase; MCAO, middle cerebral artery occlusion; MDA, Malondialdehyde; MDH, malate dehydrogenase; MIC, minimum inhibitory concentration; MLKL, mixed-line kinase domain-like protein; MMP, matrix metalloproteinase; mTOR, mammalian target of rapamycin; MW, microwave; MYC, Myelocytomatosis oncogene; NADPH, nicotinamide adenine dinucleotide phosphate; ND, neovascularization; NF-κB, nuclear factor kappa-B; NHEKs, normal human epidermal keratinocytes; NO, nitric oxide; NQO1, NAD (P) H quinone dehydrogenase 1; Nrf2, Nuclear factor (erythroid-derived 2)-like 2; NSCLC, non-small cell lung cancer; OA, oleic acid; OGD, oxygen and glucose deprivation; PacrtB, pantoea agglomerans CrtB; PARP, poly ADP-ribose polymerase; PARP-1, poly ADP-ribose polymerase 1; PEF, pulsed electric field; PEPC, phosphoenolpyruvate carboxylase; PGE2, prostaglandin E2; PI3K, phosphatidylinositol 3-kinase; PKA, protein kinase A; PPARγ, peroxisome proliferator-activated receptor γ; PR, *phaffia rhodozyma*; PV, peroxide value; PYE, *Pyropia yezoensis* extracts; RCA, ribulose bisphosphate carboxylase/oxidase (Rubisco) activator enzyme; Reg-3γ, regenerating family member gamma; RGCs, retinal ganglion cells; RIP, receptor-interacting protein kinase; RNS, reactive nitrogen species; ROS, reactive oxygen species; SAH, aneurysmal subarachnoid hemorrhage; SCII, spinal cord ischemia-reperfusion injury; SFE, supercritical fluid extraction; SFE-CO_2_, supercritical carbon dioxide; SIRT1, silent information regulator 1; SOD, superoxide dismutase; SSW, shrimp shell waste; STAT3, signal transducer and activator of transcription 3; STING, stimulator of interferon genes; STZ, streptozotocin; TA, total AST; TARC, Thymus- and Activation-Regulated Chemokine; TBARS, Thiobarbituric acid active substance; TC, total cholesterol; TFA, ethyl fatty acid; TNF-α, Tumor necrosis factor-α; T-AOC, total antioxidant capacity; Tyr-P, Tyr phosphorylation; T2DM, Type 2 diabetes; UAE, ultrasonic-assisted extraction; UVB, ultraviolet B; VEGF, vascular endothelial growth factor; VEGFR2, VEGF receptor 2; WSE, walnut shell extract; XBP1, X-box binding protein 1; *X. dendrorhous*, *Xanthophyllomyces dendrorhous*; Z-AST-D, Z-isomer AST; ZEB1, Zinc Finger E-Box Binding Homeobox 1.
